# Development of Live Attenuated *Salmonella* Typhimurium Vaccine Strain Using Radiation Mutation Enhancement Technology (R-MET)

**DOI:** 10.3389/fimmu.2022.931052

**Published:** 2022-07-11

**Authors:** Hyun Jung Ji, A-Yeung Jang, Joon Young Song, Ki Bum Ahn, Seung Hyun Han, Seok Jin Bang, Ho Kyoung Jung, Jin Hur, Ho Seong Seo

**Affiliations:** ^1^Research Division for Radiation Science, Korea Atomic Energy Research Institute, Jeongeup, South Korea; ^2^Department of Oral Microbiology and Immunology, and Dental Research Institute (DRI), School of Dentistry, Seoul National University, Seoul, South Korea; ^3^Department of Internal Medicine, Korea University College of Medicine, Seoul, South Korea; ^4^Research and Development Center, HONGCHEON CTCVAC Co., Ltd., Hongcheon, South Korea; ^5^Department of Veterinary Public Health, College of Veterinary Medicine, Jeonbuk National University, Iksan, South Korea; ^6^Department of Radiation Science, University of Science and Technology, Daejeon, South Korea

**Keywords:** live vaccine, radiation, R-MET, *Salmonella typhimurium*, mutation- genetics, attenuation

## Abstract

*Salmonella enterica* is a leading cause of food-borne diseases in humans worldwide, resulting in severe morbidity and mortality. They are carried asymptomatically in the intestine or gallbladder of livestock, and are transmitted predominantly from animals to humans *via* the fecal-oral route. Thus, the best preventive strategy is to preemptively prevent transmission to humans by vaccinating livestock. Live attenuated vaccines have been mostly favored because they elicit both cellular and humoral immunity and provide long-term protective immunity. However, developing these vaccines is a laborious and time-consuming process. Therefore, most live attenuated vaccines have been mainly used for phenotypic screening using the auxotrophic replica plate method, and new types of vaccines have not been sufficiently explored. In this study, we used Radiation-Mutation Enhancement Technology (R-MET) to introduce a wide variety of mutations and attenuate the virulence of *Salmonella* spp. to develop live vaccine strains. The *Salmonella* Typhimurium, ST454 strain (ST WT) was irradiated with Cobalt^60^ gamma-irradiator at 1.5 kGy for 1 h to maximize the mutation rate, and attenuated daughter colonies were screened using *in vitro* macrophage replication capacity and *in vivo* mouse infection assays. Among 30 candidates, ATOMSal-L6, with 9,961-fold lower virulence than the parent strain (ST454) in the mouse LD_50_ model, was chosen. This vaccine candidate was mutated at 71 sites, and in particular, lost one bacteriophage. As a vaccine, ATOMSal-L6 induced a *Salmonella*-specific IgG response to provide effective protective immunity upon intramuscular vaccination of mice. Furthermore, when mice and sows were orally immunized with ATOMSal-L6, we found a strong protective immune response, including multifunctional cellular immunity. These results indicate that ATOMSal-L6 is the first live vaccine candidate to be developed using R-MET, to the best of our knowledge. R-MET can be used as a fast and effective live vaccine development technology that can be used to develop vaccine strains against emerging or serotype-shifting pathogens.

## Introduction

Invasive non-typhoidal *Salmonella* (iNTS) is a leading cause of bacterial bloodstream infections in both humans and animals (1). *Salmonella* infections commonly result in self-limiting diarrheal illness that rarely leads to deaths; however, the case fatality rate increases to 20−25% in infants, elderly, and immunocompromised individuals (2–5). Recent systemic analysis reported that iNTS caused an estimated 535,000 illnesses and 77,500 deaths in 2017 (6), particularly in sub-Saharan Africa, where iNTS is a leading cause of community-onset bloodstream infection (7–9). In that region, iNTS was the second most common invasive bacterial disease, following *Streptococcus pneumoniae* infection (3, 7, 10). Although *Salmonella* can be controlled using antibiotics, an increased prevalence of multidrug-resistant strains has been reported over recent decades (11–13). Vaccines can potentially control the prevalence of *Salmonella* in both humans and animals (14–16). There are two possible vaccination strategies (4); vaccinating high risk groups among humans, such as elderly and/or immunocompromised adults and (5) mass vaccination to poultry and pig to prevent transmission of *Salmonella* to human *via* the consumption of *Salmonella*-contaminated meat.

Approximately 20–36% of *Salmonella* cases in humans were linked to the consumption of eggs, poultry meat, and red meats contaminated with *Salmonella* (17). At present, there is no vaccine that directly targets *S. *Typhimurium in humans, but several types of vaccines have been introduced to pigs and chickens (18–20). Surprisingly, the mass poultry vaccinations carried out in the United Kingdom, which were introduced to combat *Salmonella* infections, has dramatically decreased transmitted illness from 1.6 cases per 1000 persons in 1993 to 0.2 cases per 1,000 persons in 2009 (21). Therefore, vaccinating economically important animals might be the safest and most effective strategy to prevent the spread of *Salmonella* infection in humans.

Primarily, live attenuated vaccines have been favored because they elicit both cellular and humoral immunity, which provide long-term protective immunity (22). To date, several live attenuated vaccines are available worldwide for use in pig and poultry. In Australia, there is only one registered, commercially available live attenuated *S.* Typhimurium vaccine; it was produced by disrupting the *aroA* gene by inserting the Tn10 transposon (23). IDT Biologika licensed Salmovac440 for chickens and Salmoporc for pigs, which are auxotrophic *Salmonella* vaccine strains of both histidine and adenine (24, 25). Recently, whole genome sequencing (WGS) results showed that Salmonvac440 was attenuated by only 6 SNPs, and these mutations dramatically reduced *Salmonella* virulence (26). However, the mutations caused by SNPs easily revert and regain original virulence. To overcome this, a *Salmonella* vaccine strain using LMO (Living Modified Organism) is being developed. CVD1921, which is mutated in both the *guaBA* genes that are involved in the biosynthesis of guanine nucleotides, and the *clpP* gene affecting flagella expression, was shown to be significantly attenuated with decreased shedding, systemic spread, and clinical disease manifestations in the digestive tract of a primate model (rhesus macaque) (27). Nevertheless, LMO vaccines have not been approved for use in the farm in many countries due to environmental contamination risks and transmission of modified genes to environmental microorganisms.

Spontaneous mutations have been extensively used as sources of novel genetic diversity for selecting new, improved organisms (28, 29). However, the appearance of new mutations is a very rare event in bacteria, because the mutation rate of *Escherichia coli* is only 10^-3^ per genome per generation (30). After Hermann Joseph Muller first discovered that exposure to high-energy radiation induces a variety of genetic mutations and can transmit these new mutations to offspring (31, 32), radiation-induced mutation breeding is being widely used to generate genetic variability in various organisms (33). Radiation-induced mutagenesis can be caused by direct or indirect action on the DNA. In the direct action method, the radiation penetrates the cell and hits the DNA causing single-stranded or double-stranded DNA breaks (34). In the indirect action method, the radiation hits the water molecules, the major constituent of the cell, and other organic molecules in the cell, whereby free radicals such as hydroxyl (HO•) and alkoxy (RO•) are produced. Free radicals are characterized by an unpaired electron in the structure, which is highly reactive and reacts with DNA molecules to cause molecular structural damage (35–37). Chemical mutagens and ultraviolet rays have been widely used to accelerate the onset of mutations and develop live attenuated vaccine strains, but SNPs are the major type of mutations and deletions, and insertions are limitedly introduced in the genome (37–39). However, radiation can cause spontaneous DNA mutations including deletions, insertions, and point mutations. In fact, we first introduced radiation mutation enhancement techniques (R-MET) to induce various mutations in cancer targeting *Salmonella* in our previous study (40). However, R-MET has not yet been applied to vaccine development.

In this study, we developed a hyper-attenuated, but immunologically active *Salmonella* vaccine strain ATOMSal-L6 by accelerating mutation using gamma irradiation. ATOMSal-L6 is at least 9,961-fold less virulent than its parent strain, but can enhance both humoral and cellular immune responses, and was found to confer protective immunity in both mice and porcine models. In addition, WGS analysis showed that ATOMSal-L6 introduced many SNPs and deletion/insertion mutations. This newly developed attenuated vaccine strain is a genetically stable vaccine strain that can potentially overcome the shortcomings of existing vaccines and can be easily and quickly developed into bacterial vaccines using radiation.

## Materials and Methods

### Ethics Statement

This study was performed in strict accordance with the recommendations of the Guide for the Care and Use of Laboratory Animals of the National Institutes of Health. All animal experiments were approved by the Committee on the Use and Care of Animals at the Korea Atomic Energy Research Institute (KAERI; approval no. KAERI-IACUC-2020-004, KAERI-IACUC-2021-003) according to accepted veterinary standards set by the KAERI animal care center. Mice were euthanized by CO_2_ inhalation, as specified by the KAERI Institutional Animal Care and Use Committee guidelines.

### Bacterial Strains

*S.* Typhimurium ST454 (ST WT) was obtained from the Korea Veterinary Culture Collection (Kimchun, Republic of Korea), ATOMSal-L6 was derived from ST WT, and gene mutation was induced by gamma-radiation. Their genome was sequenced using the PacBio RS II platform (Pacific Biosciences, Menlo Park, CA, USA) and Illumina Hiseq platform at Macrogen Co., Ltd. (Seoul, Republic of Korea). The assembled genome of ST WT contained two contigs, one circular genome (4,823,318 bp) and one plasmid (109,428 bp). After complete genome assembly, BLAST analysis (v2.7.1) was carried out to identify the species to which each scaffold showed the highest similarity. The best hit was *S. enterica* subsp. *enterica* strain ST1120 (accession number: CP021909.1).

### Mutation Rate Analysis

ST WT was grown in Luria-Bertani (LB; Difco, BD Biosciences, Franklin Lakes, NJ, USA) broth at 37°C and 200 rpm under aerobic conditions. The stain attained an optical density (OD_600_) of 0.5, exposed to 0.5–3.5 kGy for 1 h at room temperature using a ^60^Co-gamma irradiator (point source AECL, IR-79, MDS Nordion International Co., Ottawa, Canada) at the Advanced Radiation Technology Institute of KAERI (Jeongeup, Republic of Korea). The irradiated samples were concentrated and plated on LB agar plates with 10 μg/mL of kanamycin to select for mutants that acquired kanamycin resistance, as has been described previously (41). The overall mutation rate of the population was calculated using the mean number of mutants.

### Macrophage Invasion and Replication Assay

RAW 264.7 cells were purchased from the American Type Culture Collection (ATCC, Manassas, VA, USA), and was grown in high-glucose Dulbecco’s modified Eagle’s medium (DMEM; Sigma-Aldrich; St. Louis, MO, USA) supplemented with 10% fetal bovine serum (FBS; Biowest, Nuaillé France), and 1% antibiotics (100 U/mL penicillin and 100 μg/mL streptomycin; Gibco; Waltham, MA, USA) at 37°C in the presence of 5% CO_2_. RAW 264.7 cell (3 x 10^4^ cells per well) was seeded on 48-well plates (SPL Life Sciences, Pocheon, Republic of Korea) and incubated for 16 h. Attenuated *Salmonella* candidates were cultured in LB at OD_600_ of 1.0 and harvested. The strains were treated to RAW 264.7 cells at multiplicity of infection (MOI) of 10 and incubated for 2 h at 37°CC. The cells were washed with PBS (Welgene, Gyeongsan, Republic of Korea) thrice and transferred to DMEM supplemented with 10% FBS and 100 μg/mL of gentamicin to eliminate extracellular bacteria. After 2 h incubation, the cells were washed three times with PBS and treated with RIPA lysis buffer (Sigma) for invasion assay. For replication assay, the cells were incubated with DMEM supplemented with 10% FBS and 10 μg/mL gentamicin to prevent the leakage of intracellular bacteria. After 16 h incubation, cells were treated with RIPA lysis buffer (Sigma). Subsequently, lysis samples were serially diluted with PBS and spotted on the LB agar plate.

### Biochemical Characteristics Analysis

To compare bacterial growth rates with temperature (37°C, 42°C, and 45°C), overnight cultured ST WT and ATOMSal-L6 were re-inoculated into 150 mL LB medium and the bacterial OD_600_ was calculated every 30 min. Overnight cultured ST WT and ATOMSal-L6 were re-inoculated into 20 mL LB medium at OD_600_ of 1.0. Biochemical features of ST454 and ATOMSal-L6 were analyzed using the API ZYM (enzyme activities), API 20NE, and API 50CH (utilization of carbohydrate) kits (bioMerieux, Inc.; Marcy L Etoile, France) according to manufacturer’s instructions. In brief, the cultured bacteria were diluted using the provided medium until adequate turbidity was attained. Diluted samples were added into the cupules, and incubated for 48 h at 37°C.

### Antimicrobial Susceptibility Test (MIC Test)

The MIC test was measured the antimicrobial susceptibilities of ST WT and ATOMSal-L6 to kanamycin (KAN, 50 mg/mL), tetracycline (TET, 50 mg/mL), erythromycin (ERM, 150 mg/mL), ampicillin (AMP, 100 mg/mL), Cefadroxil (CFR, 1 mg/mL), trimethoprim (TMP, 25 mg/mL), gentamicin (GEN, 0.5 mg/mL), amoxicillin (AMC, 1 mg/mL), amikacin (AMK, 1 mg/mL), streptomycin (STR, 1 mg/mL), spectinomycin (SPT, 1 mg/mL), lincomycin (LIN, 1 mg/mL), clindamycin (CLI, 1 mg/mL), and tobramycin (TOB, 1 mg/mL). Briefly, overnight cultured ST WT and ATOMSal-L6 were re-inoculated into 30 mL LB medium (1:50 dilution), 100 μL of diluted sample was added into round bottom 96 well plate (SPL) with 3-fold diluted antibiotics.

### Scanning Electron Microscope Analysis

ST WT and ATOMSal-L6 were fixed with 4% glutaraldehyde solution at 4°C and kept overnight. After centrifuging, the fixed samples were washed thrice with PBS and dehydrated using 30, 50, and 70% ethanol sequentially, following which the samples were dried and coated with gold sputter. The plate was observed using a JEIL JSM-840 Scanning Microscope (Tokyo, Japan) at the Seoul National University.

### Motility Assay

Motility medium, which was composed of LB supplemented with 0.4% agar (BD) and 1% triphenyltetrazolium chloride (TTC; Sigma) was poured into the 14 mL round bottom tube (SPL). Overnight cultured ST WT and ATOMSal-L6 were re-inoculated into 3 mL LB medium at an OD_600_ of 1.0. The cultured samples were pierced deeply into the motility medium using the loop (SPL). The tubes were incubated for 3 days at 37°C.

### High-Throughput Sequencing and Comparative Genomic Analysis

The location of nucleotide substitutions, deletions, and insertions in the genome of the attenuated strain ATOMSal-L6 was determined using Illumina HiSeq 2000 (150 bp paired-end) with 825.98–fold coverage. The total length of read bases was 4,088,887,030 bp, which covered 99.98% length of the ST WT strain. The raw reads from the ST WT genome were mapped and aligned to the reference genome sequence using Burrows-Wheeler aligner (BWA-0.7.12) and Picard. Next, the genetic variants were detected using SAMTools (ver. 1.2). All coding variants were identified based on the open reading frames of ST WT. The whole-genome sequences of ST WT (ST454) and ATOMSal-L6 has been deposited in DDBJ/EMBL/GenBank under the accession number CP098438-CP098439. The BioProject accession numbers are PRJNA844490, and PRJNA841760 and the BioSample accession number are SAMN28818465 and SAMN28614156, respectively.

### Mouse and Pig Experiments

The animal housing conditions, which were designed for specific pathogen-free animals, and the animal experimental design were approved by the Committee on the Use and Care of Animals at the KAERI and implemented according to the ethical standards accepted by the National Health Institute. The ventilated housing cage (Orient Bio Inc., Seoul, Republic of Korea) was maintained in an animal biological safety level 2 facility at 22–23°C on a 12 h:12 h light:dark cycle. The cages were covered with high-efficiency particulate air-filtered micro-isolation lids (Orient Bio Inc.) in a static airflow environment. Bedding (Beta Chip; Orient Bio Inc.) at an approximate depth of 1.0 cm was changed weekly. Irradiated rodent diet food (5053; Orient Bio Inc.) and sterile water were provided *ad libitum* through a wire cage top. Five-week-old male C57BL/6 or BALB/c mice (weight 19–21 g) were purchased from Orient Bio Inc. Five C57BL/6 mice were randomly assigned to individually ventilated housing cages and immunized i.m. or orally thrice at two-week intervals with either PBS, ATOMSal-L6 (10^5^, 10^6^, 10^7^, 10^8^ CFU/mouse) strain vaccine. No significant weight loss, mortality, or serious clinical signs were observed after vaccination. Two weeks after the third vaccination, blood was collected to measure ST-specific antibodies, and the spleen was collected to measure ST-specific T cell responses. To examine the protective efficacy of the vaccination, mice were challenged i.p. with ST WT (5 × 10^5^ CFU/mouse) two weeks after the third vaccination. Mouse survival was monitored for 14 days.

All pigs were acclimatized according to the protocols of the Central Animal Research Laboratory at the Chonbuk National University (Iksan, Republic of Korea). Pregnant sows were divided equally into two groups (n=3). All groups were primed orally during week 8 of pregnancy and boosted orally during week 11 of pregnancy with approximately 2 × 10^9^ CFU of ATOMSal-L6. Blood samples were collected from the pregnant sows in all groups as the same methods mentioned in the previous study (42) at 0 (prior to priming during week 8 of pregnancy), 3 (prior to the booster during week 11 of pregnancy), 5 and 8 (on the day of farrowing) weeks post prime immunization (PPI). Colostrum samples were collected from the sows within 4 h after farrowing. In addition, blood samples were taken from the jugular veins of their suckling piglets 6 days after birth. Three weeks after the second vaccination, the sows and piglets were challenged orally with ST WT (5 × 10^8^ CFU). Survival was monitored for 14 days.

### Measurement of *Salmonella*-Specific Immunoglobulin Levels

Blood samples from mice and pigs were obtained 14 days after the last vaccination. *Salmonella* antigen lysates were prepared as was elaborated in previous sections. ST WT was grown in LB broth and harvested at OD_600_ = 0.8. The pellet was washed with PBS followed by sonication 30 times for 5 s. Samples were centrifuged at 13,000 rpm for 10 min at 4°C, and the supernatants were collected and stored at –70°C. Total protein concentration was measured using the Pierce™ BCA Protein Assay Kit (Thermo Fisher Scientific, Waltham, MA, USA). To examine the levels of ST-specific immunoglobulins (Igs), *Salmonella* lysate (10 μg/well) was immobilized on 96-well plates for 16 h at 4°C, followed by blocking with 1% BSA in PBS. After washing thrice with PBS containing 0.05% Tween-20 (PBS-T; Sigma-Aldrich), serial two-fold dilutions of mouse or pig serum (100 μL) were added to each well and incubated at 23°C for 2 h. The plates were washed five times with PBS-T to remove unbound antibodies, and bound antibodies were detected using horseradish peroxidase (HRP)-conjugated anti-mouse Igs (anti-mouse IgM, IgG, IgG1, and IgG2a; 1:5000 dilution in PBS-T; Sigma-Aldrich) or HRP-conjugated anti-pig IgM and IgA (1:5000 dilution in PBS-T; Southern Biotech, Birmingham, AL, USA) for 1 h at room temperature. After washing seven times with PBS-T, 100 μL of 3,3’,5,5’-tetramethylbenzidine substrate solution (INTRON Inc., Seoul, Republic of Korea) was added, followed by incubation for 5–10 min at 23°C. When the color was sufficiently developed, 50 μL of 2 N H_2_SO_4_ stop solution (Daejung Chemicals; Siheung, Republic of Korea) was added. The absorbance at 450 nm was measured using an Epoch 2 plate reader (BioTek).

### Splenocytes Analysis by Flow Cytometry

Two weeks after the final immunization, spleens from mice immunized with either the PBS or ATOMSal-L6 vaccine were isolated and filtered through a cell strainer (70 µm; SPL). Red blood cells (RBCs) were lysed with RBC lysis buffer (Sigma-Aldrich) and washed with RPMI-1640 medium containing 10% FBS. The cell suspension was seeded into a 48-well plate (2 × 10^6^ cells/well) and stimulated with 10 µg/mL ST WT lysate, 0.5 µg/mL GolgiStop (BD Bioscience, San Diego, CA, USA), and 0.5 µg/mL GolgiPlug (BD Bioscience) at 37°C for 12 h. To analyse Helper T cells, the cells were washed with PBS and stained with a Live/Dead Staining Kit (L/D; *In vivo*Gen, San Diego, CA, USA), anti-CD8-FITC (BD Bioscience), and anti-CD4-BV421 (BD Biosciences) for 20 min at 23°C to stain T cell surface markers. Cells were fixed and permeabilized using a Cytofix/Cytoperm kit (BD Bioscience) for 20 min at 4°C, and the intracellular cytokines were stained with anti-IFN-γ-PE (BD Biosciences), anti-IL-5-APC (BD Bioscience), and anti-IL-17A-PE-Cy7 (BD Bioscience) for 20 min at 23°C. After staining, the cells were analyzed using a MACS Quant flow cytometer (Miltenyi Biotec, San Diego, CA, USA) and FlowJo software (TreeStar, Ashland, OR, USA). For further analysis of the multifunctional T cells, the staining was performed in the same method as was described above. Briefly, the T cells surface staining antibodies were used with 7-AAD (7-Amonoactinomysin D; Sigma), anti-CD3e-Alexa Fluro 488 (BD Biosciences), anti-CD4-BV421 (BD Biosciences), and anti-CD8-V500 (BD Biosciences) and intracellular cytokines staining antibodies were stained with anti-IFN-γ-PE (BD Biosciences), anti-TNF-α-APC (BD Bioscience), and anti-IL-2-PE-Cy7 (BD Bioscience).

### Adoptive Transfer of Sera, CD4^+^ or CD8^+^ T Cells

Individual mouse sera, prepared as described above, were mixed and 100 μL of pooled sera were administrated i.p. to naïve C57BL/6 mice (n = 5). Mouse spleen cells were prepared by passing spleen specimens through a cell strainer (70 µm; SPL), and red blood cells were lysed with RBC lysis buffer (Sigma-Aldrich). Splenic CD4^+^ and CD8^+^ T cells were separated using Miltenyi MACS microbeads conjugated with anti-CD4 and anti-CD8 monoclonal antibodies (Miltenyi Biotec) and a MACS LS column (Miltenyi Biotec). Isolated CD4^+^ or CD8^+^ T cells (5 × 10^6^ cells or 5 × 10^5^ cells/mouse) were administered i.p. to naïve C57BL/6 mice (n = 5). After 12 h, mice were challenged i.p. with ST WT (5 × 10^5^ CFU/mouse) and mouse survival was monitored for 14 days.

### Statistical Analysis

Data are expressed as the mean ± standard deviation (SD). Data in the bar and dot graphs between groups were compared using an unpaired Student’s *t*-test for normal data distribution or the Mann–Whitney non-parametric test for abnormal data distribution using GraphPad Prism (version 7.0; GraphPad Software, Inc., La Jolla, CA, USA). The survival of mice was determined using Kaplan–Meier survival analysis, and the significance of the difference was analyzed using a log-rank test with GraphPad Prism software. P < 0.05 was considered statistically significant.

## Results

### Construction of the Attenuated *Salmonella* Strain (ATOMSal-L6) Using R-MET

**Figure 1:**
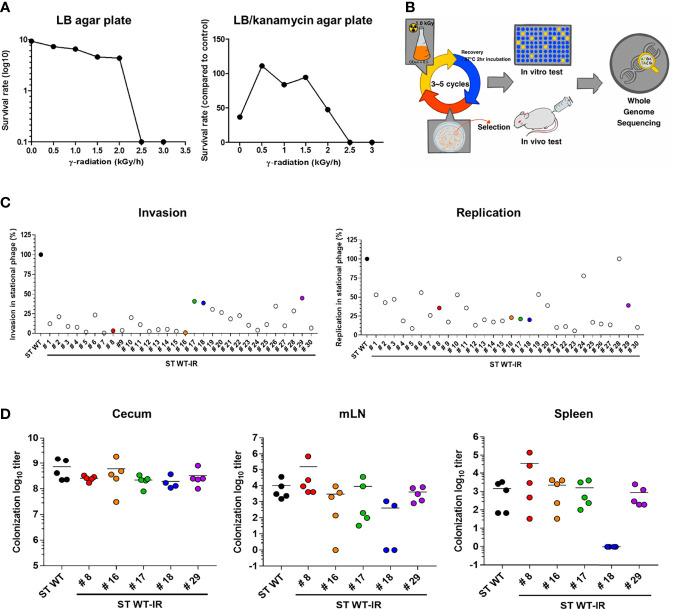
Construction of attenuated *Salmonella* vaccine strain using radiation mutation enhancement techniques (R-MET). **(A)** The radiation dose selection for R-MET. ST WT was irradiated with high-dose ^60^Co gamma-ray. The ratio of survived/dead bacteria was evaluated by plating on LB plate (left panel) and mutated strains were detected by plating on LB plate with 10 μg kanamycin (right panel). **(B)** Schematic for developing attenuated *Salmonella* vaccine strains. **(C)** *In vitro* virulence test. Macrophages (RAW 264.7 cells) were infected with ST WT or selected candidates (n=30) at a multiplicity of infection of 10 and bacterial viability was evaluated at 2 h (invasion; left panel) or 18 h (replication; right panel). Relative invasion and replication fold of candidates were calculated by comparing with ST WT. Data are representative of three independent experiments and are presented as the mean ± standard deviation. **(D)** *Salmonella* colonization in mice organs. BALB/c mice (n=5) were inoculated orally with 1×10^8^ CFU WT and attenuated candidates (8, 16–18, 29). At 72 hpi, the number of bacteria in the cecum (left panel), mLN (middle panel), and spleen (right panel) were counted. Data were presented as the mean ± standard deviation. CFU, colony forming unit; WT, wild type; hpi, hours post-infection; mLN, mesenteric lymph nodes.

Radiation mutatin rate was calculated as the rate of generation of antibiotic resistant before and after irradiation (43). To optimize the R-MET condition, ST WT (10^9^ - 10^10^ CFU, A_600_ = 1.0) was irradiated with the indicated dose of gamma ray and then plated on LB agar with or without kanamycin. As was shown in **Figure 1A**, the number of ST WT on the LB agar plate gradually decreased after irradiation, and no colonies were detected above a radiation dose of 2.5 kGy. In contrast, kanamycin-resistant mutations were not detected before irradiation, but were predominantly present at doses between 0.5–1.5 kGy. We compared the ratio of survived viable and mutated bacteria and selected 1.5 kGy as the optimal radiation dose, because it gave rise to 0.88 ± 0.18 mutants/10^10^ CFU. A schematic procedure for the development of an attenuated *Salmonella* vaccine strain is presented in **Figure 1B**. To construct an attenuated vaccine strain, the ST WT strain was exposed to 1.5 kGy γ-radiation for 1 h followed by plating on LB agar. After incubation for at least 2 days at 28°C, unusual shaped colonies were picked and inoculated into LB broth. This process was repeated 3 or more times to enrich the mutated strains. Finally, 30 colonies were selected as the mutant candidates of ST WT.

The ability of *Salmonella* to invade and replicate in the intracellular vacuoles is crucial for the initial stage of an invasive disease (44). Therefore, we examined the attenuation of mutant candidates by performing cell invasion and replication assays and compared them to the ST WT. RAW264.7 monolayers were infected with each mutant strain (ST WT-IR #) and invasion (2h) and replication (18h) rates were compared to the ST WT strain (**Figure 1C**). Most of the selected mutants showed at least 50% lower levels of invasion and replication capacity than the parent strain (ST WT). We selected five mutants. The mutants #8 and #16 had lower levels of invasiveness (<1%), but higher levels of replication (>25%). Mutants #17 and #18 had high levels of invasiveness (>40%), but low levels of replication (<25%). Mutant #29 was chosen as the control mutant.

To compare virulence, mice (BALB/c; n=5/group) were orally inoculated with the candidates (ST WT-IR #8, #16, #17, #18, #29) and their colonization in cecum and invasion into the spleen and mesenteric lymph node (mLN) were counted 1 day post infection (d.p.i.). Compared to the ST WT, most of the mutants, except #18, had similar levels of colonization in the caecum, spleen, and mLN. Mutant #18 did not show significant change in the level of colonization in the cecum, but showed a significant reduction in organ invasiveness compared to the ST WT (**Figure 1D**). No bacteria were detected in the blood, liver, and lungs (data not shown). Thus, #18 was possibly the most attenuated mutant among the selected candidates. To analyze the lethal dose 50 (LD_50_), mice (n=3/group) were injected with an increasing dose of #18 or ST WT i.p. LD_50_ was calculated using “Quest Graph LD_50_ calculator”, ST WT was 2.71 × 10^4^ CFU/mouse, while #18 was approximately 2.69 × 10^8^ CFU/mouse, making #18 about 9,961 times less virulent than its parent strain (ST WT); therefore, #18 was designated ATOMSal-L6 in this study.

### Genetic and Biochemical Characterization of ATOMSal-L6

**Figure 2:**
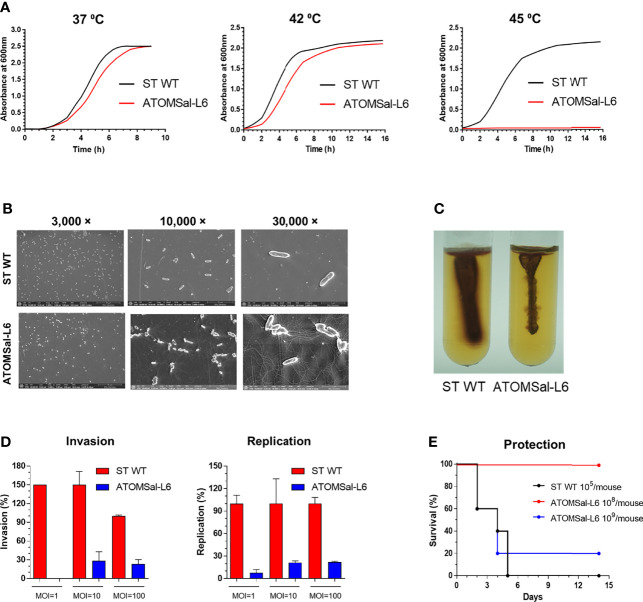
Analysis of biochemical and genetic characteristics of ATOMSal-L6. **(A)** Growth curve of ST WT and ATOMSal-L6 at 37°C, 42°C, and 45°C. **(B)** Morphology analysis of ST WT and ATOMSal-L6 using scanning electron microscopy (SEM; left panel). **(C)** *Salmonella* motility assay for ST WT and ATOMSal-L6 on LB containing 0.4% agar and 1% triphenyltetrazolium chloride at 37°C for 3 days. One *Salmonella* colony on LB agar plate was stabbed once to a depth of ~2 cm in the middle of the motility agar tube (right panel). **(D)** *In vitro* virulence test. Macrophages (RAW 264.7 cells) were infected with ST WT or selected candidates at MOI = 1, 10, 100, and bacterial viability was evaluated at 2 h (invasion; left panel) or 18 h (replication; right panel). Relative invasion and replication fold of candidates were calculated by comparing with ST WT. Data are representative of three independent experiments and are presented as the mean ± standard deviation. **(E)** *In vivo* virulence of ATOMSal-L6. C57BL/6 mice (n=5) were infected i.p. with ST WT (10^5^ CFU) or ATOMSal-L6 strain (10^8^, 10^9^ CFU) and survival was monitored for 14 days.

To confirm the phenotypical stabilization of ATOMSal-L6 strain, it was sequentially cultured 10 times in LB broth and re-examined for virulence. When growth rates were compared with ST WT, ATOMSal-L6 showed a similar growth pattern to ST WT at 37°C and 42°C, but no growth at 45°C (**Figure 2A**). Next, we examined its biochemical characteristics using Analytical Profile Index (API) analysis (**Tables S1–3**). The biochemical profiling of Gram-negative identification (API 20NE) showed no differences; however, esterase (C4) and several carbohydrates utilization profiled (API ZYM, 50CH) were slightly different compared to ST WT. For example, ATOMSal-L6 fully utilized esterase and carbon sources (L-arabinose and D-mannose) and showed weak signal at D-ribose, L-rhamnose, and melibiose, but ST WT did not. We tested the antibiotic susceptibility of ST WT and ATOMSal-L6 with MIC (**Table S4**). The MIC of ATOMSal-L6 against KAN, CFR, TMP, and TOB were same with ST WT. The MIC of ATOMSal-L6 against TET, ERM, GEN, AMC, AMK, and STR were 3-fold lower than ST WT. The MIC of ATOMSal-L6 against AMP, SPT, LIN, and CLI were more than 9-fold lower than ST WT. These data indicated that ATOMSal-L6 likely loses its ability to resist antibiotic stress during R-MET process.

To directly visualize the extracellular structure of ATOMSal-L6, Scanning Electron Microscopy (SEM) was performed (**Figure 2B**). Compared with ST WT, ATOMSal-L6 showed no significant difference in size and shape; however, a higher level of flagellin was expressed (**Figure 2B**). To examine whether higher expression of flagellin affected the motility of ATOMSal-L6, we performed swarming assay (Surprisingly, even though ATOMSal-L6 expressed higher flagellin than ST WT, its motility on semi-solid swarming agar media was generally lower than that of ST WT (**Figure 2C**).

The virulence attenuation of ATOMSal-L6 was re-examined *in vitro* and *in vivo*. ATOMSal-L6 or ST WT was added onto RAW 264.7 monolayers at MOIs of approximately 1, 10, or 100 and their invasion and replication abilities were compared as above (**Figure 2D**). As expected, ATOMSal-L6 showed dramatically reduced invasiveness and replication capacity compared to ST WT. When mice (n=5/group) were injected i.p. with ST WT or ATOMSal-L6, all mice infected with ST WT (10^5^ CFU/mice) died within 5 days post-infection and only 20% of mice were survived by infecting with extremely high number of ATOMSal-L6 (10^9^ CFU/mouse), whereas all mice infected with 1,000-fold higher numbers of ATOMSal-L6 (10^8^ CFU/mouse) exhibited 100% survival for more than 14 days (**Figure 2E**).

To analyze the location of mutations in ATOMSal-L6, the complete genome of ATOMSal-L6 was sequenced and compared to ST WT as a reference genome. As shown in **Figure S1A**, we found 137 mutations in ATOMSal-L6 genome, including 6.56% (n=9) of point mutation (transition and transversion), 90.51% (n=124) of insertion, and 2.92% (n=4) of deletion. Mutation sites were designated to the circular form of ATOMSal-L6 genome (**Figure S1B** and **Table S5**). Surprisingly, only 9 mutations were occurred in A or T nucleotides and the others (n=126) were all mutated in G and T. Of note, we found that ATOMSal-L6 lost one bacteriophage located at 3,440,538 bp - 3,481,579 bp encoded by IS1595 transposase phage genes (gene bank number = CP098438-CP098439). All these data suggested that R-MET introduced many mutations and that these mutations could attenuate its virulence *in vitro* and *in vivo*.

### High Immune Response by Immunizing I.M. With ATOMSal-L6 Vaccine in Mice

**Figure 3:**
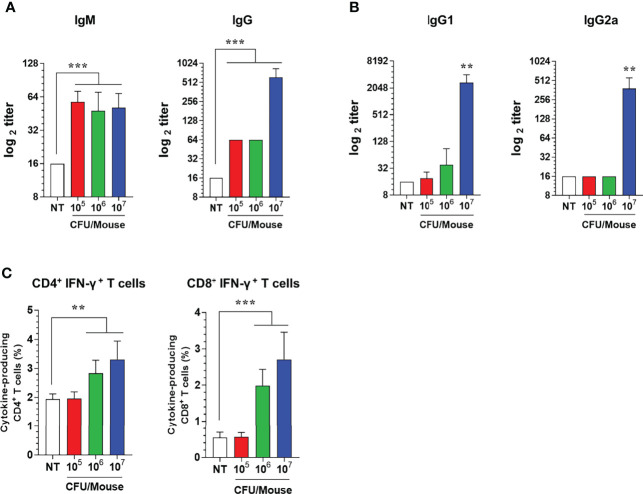
Humoral and cellular immune responses induced by i.m. immunization of ATOMSal-L6. C57BL/6 mice (n = 5 per group) were immunized i.m. with 10^5^, 10^6^, or 10^7^ CFU of ATOMSal-L6 thrice at two-week intervals. **(A, B)** Humoral immune response. Serum levels of *Salmonella*-specific IgG and IgM were analyzed at 7 days following the last immunization **(A)**. Subclass levels of *Salmonella*-specific IgG1 and IgG2a were analyzed at 7 days subsequent to the last immunization **(B)**. Data are representative of three independent experiments and are presented as the mean ± standard deviation. **(C)** Cellular immune response. Single cell suspensions of spleen were re-stimulated with 10 μg/mL ST WT lysate for 12 h and ST-specific CD4^+^ and CD8^+^ T cells were analyzed. Percentages of activated CD4^+^ and CD8^+^ T cells in spleens of vaccinated mice. Data were presented as mean ± standard deviation. *P < 0.05, **P < 0.01, ***P < 0.001, compared to unvaccinated mice.

To determine whether the ATOMSal-L6 could be used as a live attenuated vaccine, the vaccine efficacy of ATOMSal-L6 was examined using a mouse model. Mice (n=5/group) were immunized intramuscularly (i.m.) with 10^5^, 10^6^, or 10^7^ CFU of ATOMSal-L6 and *Salmonella*-specific humoral, cellular, and protective immune response were measured. At 2 weeks after the last immunization, *Salmonella*-specific IgM and IgG were measured with ELISA. As shown in **Figure 3A**, Salmonella-specific IgM was significantly increased in all groups, whereas Salmonella-specific IgG was significantly increased only in the group immunized with 10^7^ CFU compared to unvaccinated (NT) group. Furthermore, we found that Th2 response (IgG1) was the dominant immune response over Th1 (IgG2a) in the group immunized with 10^7^ CFU (**Figure 3B**).

Next, since both CD4^+^ and CD8^+^ T cells are crucial for protection against *Salmonella* infection (45, 46), we evaluated T cell subtypes induced by ATOMSal-L6 vaccination. Mice (n=5/group) were immunized i.m. thrice at 2-week intervals, and single cell splenocytes were re-stimulated with 10 μg of ST WT lysate, followed by analyzing Th1 (IFN-γ-producing CD4^+^ T cells), Th2 (IL-5-producing CD4^+^ T cells), Th17 (IL-17A-producing CD4^+^ T cells), and activated CD8^+^ T cells (IFN-γ-producing CD8^+^ T cells) using flow cytometry gating, as shown in **Figure S2**. The population of Th2 and Th17 cells was not changed after immunization (data not shown), but significant enhancement of Th1 (IFN-γ^+^ CD4^+^ T cells) and CD8^+^ T cells (IFN-γ^+^ CD8^+^ T cells) were detected when immunized with 10^6^ or 10^7^ CFU ATOMSal-L6 vaccination compared to the NT group (**Figure 3C**).

**Figure 4:**
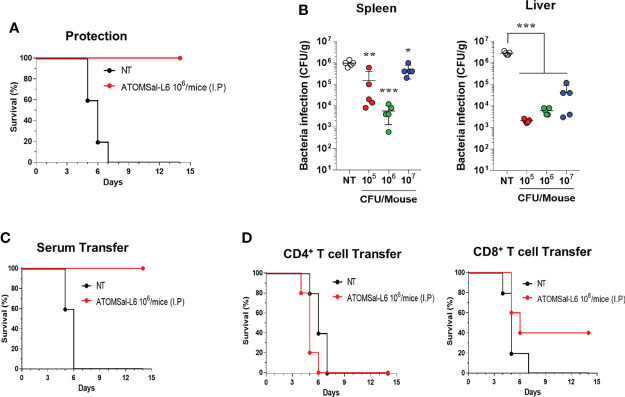
Protective immune responses induced by I.M. immunization of ATOMSal-L6. **(A, B)** Protective immune response. ATOMSal-L6 immunized mice (n=5) were challenged i.p. with 5 × 10^5^ CFU ST WT strain and survival was monitored for 14 days **(A)**. Protection of *Salmonella* colonization in mice organs by ATOMSal-L6 vaccination were measured by counting the number of bacteria in the spleen (left panel), and liver (right panel) **(B)**. Data were presented as mean ± standard deviation. *P < 0.05, **P < 0.01, ***P < 0.001, compared to unvaccinated mice. **(C, D)** Protection by the adopted transfer of ATOMSal-L6-vaccinated mice serum **(C)** and CD4^+^ or CD8^+^ T cells **(D)** against *Salmonella* infection. Mice were immunized with ATOMSal-L6 thrice at 14-day intervals. Serum (100 μL) or splenic CD4^+^ (5 × 10^6^ cells) or CD8^+^ T cells (5 × 10^5^ cells) from NT or ATOMSal-L6-immunized mice were transferred i.p. into naïve C57BL/6 mice (n=5). At 12 h following inoculation, mice were challenged intraperitoneally with 5 × 10^5^ CFU ST WT strain. Mouse survival was monitored for 14 days.

To investigate whether humoral and cellular immunity induced by ATOMSal-L6 vaccination could provide a protective immune response, ATOMSal-L6 (10^6^ CFU) vaccinated mice (n=5/group) were infected i.p. with ST WT (5 × 10^5^ CFU/mouse) and their survival monitored for 14 days. As shown in **Figure 4A**, all unvaccinated mice died at 7 d.p.i, whereas all vaccinated mice survived more than 14 days. In addition, ST WT that invaded the spleen or liver were counted at 1 d.p.i (**Figure 4B**). More than 10^6^ CFU/g of invasive bacteria were detected in the spleen and liver from unvaccinated mice, whereas significantly lower number of ST WT were detected in ones from ATOMSal-L6 vaccinated mice.

To test whether the protective immune response was due to humoral or cellular immune responses, sera (100 μL/mouse), CD4^+^ T cells (5 × 10^6^ cells/mouse), or CD8^+^ T cells (5 × 10^5^ cells/mouse) were collected from ATOMSal-L6 vaccinated, or unvaccinated mice followed by adopted transfer to naïve mice (n=5/group). After infecting i.p. with ST WT (5 × 10^5^ CFU), all mice transferred with sera or T cells from unvaccinated mice had died at 6–7 d.p.i, whereas all mice transferred with sera from ATOMSal-L6 vaccinated mice survived for more than 14 d.p.i (**Figure 4C**). Although only 40% of the mice that were provided with CD8^+^ T cells from ATOMSal-L6 vaccinated mice survived, it was not significant, but still marginally higher (p=0.1338) than that of mice transferred with CD8^+^ T cells from unvaccinated mice (**Figure 4D**). We did not observe a significant difference between CD4^+^ T cells adopted transferred from different groups (data not shown). All these data suggested that ATOMSal-L6 provided an effective immune response to protect from *Salmonella* infection by activating both humoral and cellular immune responses.

### High Immune Response by Immunizing Orally With ATOMSal-L6 Vaccine in Mice

**Figure 5:**
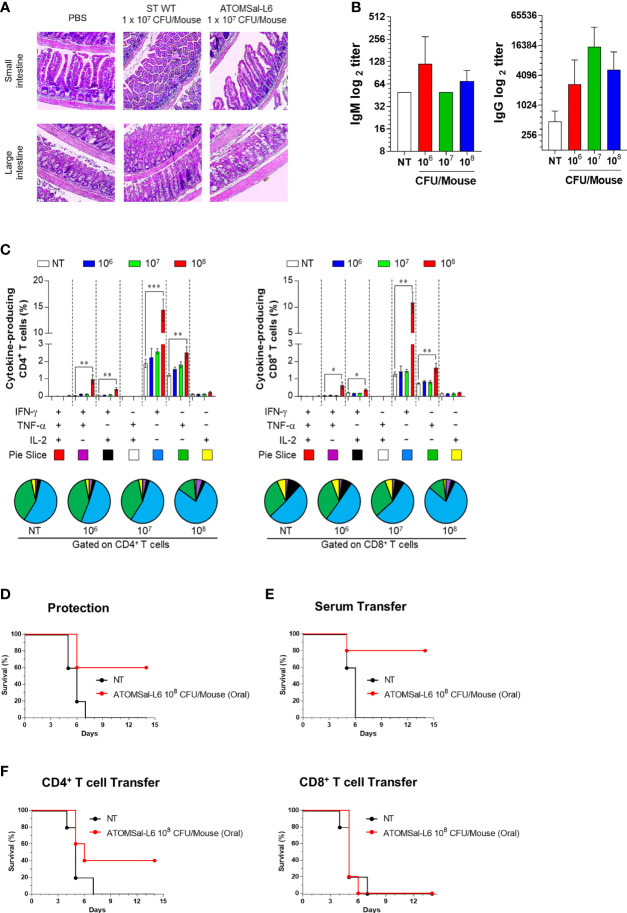
Analysis of humoral and cellular immune responses induced by oral immunization of ATOMSal-L6. **(A)** Safety of ATOMSal-L6 vaccine. Mice were inoculated orally with 10^7^ CFU of ST WT or ATOMSal-L6. At 3 d.p.i, mice were sacrificed, and small or large intestinal tissue were stained with H&E. **(B)** Humoral immune response. Mice (n = 5 per group) were orally immunized with 10^6^, 10^7^, or 10^8^ CFU of ATOMSal-L6 thrice at two-week intervals and sera were collected two weeks after the last vaccination. ST-specific IgM (left panel) and IgG (right panel) were measured using ELISA. **(C)** Cellular immune response. Single cell suspensions of spleen were re-stimulated with 10 μg/mL ST WT lysate for 12 h and ST-specific multifunctional CD4^+^ and CD8^+^ T cells were analyzed. The percentage of ST-specific total cytokine (IFN-γ, TNF-α, and/or IL-2)-producing cells among splenic CD4^+^CD44^+^ memory T cells (left panel) or CD8^+^CD44^+^ memory T cells (right panel). Pie charts (bottom panel) representing the mean frequencies of cells co-expressing IFN-γ, TNF-α, and/or IL-2. The relative amounts of single-, double-, and triple-functional memory T cells are indicated as pie arcs. Means ± SD (n = 5 mice/group) shown are representative of two independent experiments. *P < 0.05, **P < 0.01, ***P < 0.001, compared to unvaccinated mice. **(D)** Protective immune response. Mice (n=5) were immunized orally with ATOMSal-L6 (1 × 10^8^ CFU) and challenged i.p. with 5 × 10^5^ CFU of ST WT strain and survival was monitored for 14 days. **(E, F)** Protection by the adopted transfer of oral ATOMSal-L6-vaccinated mice serum **(E)** and CD4^+^ or CD8^+^ T cells **(F)** against *Salmonella* infection. Mice were immunized orally with ATOMSal-L6 twice at 14-day intervals. Serum (100 μL) or splenic CD4^+^ (5 × 10^6^ cells) or CD8^+^ T cells (5 × 10^5^ cells) from NT or ATOMSal-L6-immunized mice were transferred i.p. into naïve C57BL/6 mice (n=5). At 12 h following inoculation, mice were challenged intraperitoneally with 5 × 10^5^ CFU ST WT strain. Mouse survival was monitored for 14 days.

Because oral vaccination of live attenuated *Salmonella* vaccine is recommended for adult pigs and humans (47, 48), we next investigated whether ATOMSal-L6 could be used as an oral vaccine. To examine the virulence of ATOMSal-L6 *via* oral vaccination, mice were immunized orally with ST WT or ATOMSal-L6. No mice died even after oral administration of 10^7^ CFU of ST WT or ATOMSal-L6 (data not shown). When intestinal inflammation after ST WT infection, we observed substantial infiltration of immune cells in both the small and large intestine in the ST WT-immunized group (**Figure 5A**). No significant inflammation or damage were observed in the intestinal tissues of ATOMSal-L6 immunized mice, which showed similar results to those observed in the NT group (**Figure 5A**). To evaluate whether oral immunization of ATOMSal-L6 elicited *Salmonella*-specific immune response, mice (n=5/group) were immunized orally thrice with 10^6^, 10^7^, or 10^8^ CFU of ATOMSal-L6, and the humoral and cellular immune responses were evaluated. Oral ATOMSal-L6 vaccination resulted in a increase in serum *Salmonella*-specific IgG, and a slight increase in *Salmonella*-specific IgM (**Figure 5B**).

We next analyzed the functional composition of *Salmonella*-specific single- or multi-functional cellular immune responses (49). Mice (n=5/group) were immunized orally thrice at two-weeks interval, following which they were analyzed for *Salmonella*-specific splenic CD4^+^ T cells and CD8^+^ T cells using cytometric gating, as shown in **Figure S3**. Only the 10^8^ CFU ATOMSal-L6 were found to have significantly increased frequencies of IFN-γ^+^CD4^+^ (compared to NT group; up to 7.60-fold, p<0.001) and TNF-α^+^CD4^+^ (compared to NT group; up to 2.05-fold, p=0.005), but no changes were found from IL-2^+^CD4^+^ T cells. In addition, we found that multifunctional IFN-γ^+^ TNF-α^+^CD4^+^ (compared to NT group; up to 18.15-fold, p=0.007) and IFN-γ^+^ IL-2^+^CD4^+^ (compared to NT group; up to 6.06-fold, p=0.004) were significantly increased upon oral vaccination. Similarly, single- and multi-functional CD8^+^T cells were significantly increased in the 10^8^ CFU ATOMSal-L6 (**Figure 5C**). All these data indicated that oral immunization of live ATOMSal-L6 could induce *Salmonella*-specific humoral and cellular immunities.

To evaluate the protective immunity of ATOMSal-L6 oral vaccination, mice (n=5/group) were immunized orally thrice with ATOMSal-L6 (10^8^ CFU) at two-weeks interval, followed by injecting i.p. ST WT (ST454; 5 × 10^5^ CFU). As shown in **Figure 5D**, all unvaccinated mice died at 14 d.p.i, but 60% of the vaccinated mice survived for more than 14 d.p.i. To examine whether the protective immune response was due to humoral or cellular immune responses, sera (100 μL/mouse), CD4^+^ T cells (5 × 10^6^ cells/mouse), or CD8^+^ T cells (5 × 10^5^ cells/mouse) were collected from ATOMSal-L6 vaccinated or unvaccinated mice, followed by adopted transfer to naïve mice (n=5/group). After infecting with ST WT (5 × 10^5^ CFU), all mice transferred with sera or T cells from unvaccinated mice died at 6 - 7 d.p.i whereas 80%, 40%, and 20% of the mice transferred with sera (**Figure 5E**), CD4^+^ T cells, and CD8^+^ T cells (**Figure 5F**), respectively. All these data suggested that oral live ATOMSal-L6 vaccine provided effective immune response to protect from *Salmonella* infection by activating both humoral and cellular immune responses.

### High Protective Immune Response by Immunizing I.M. With ATOMSal-L6 Vaccine in Pig

**Figure 6:**
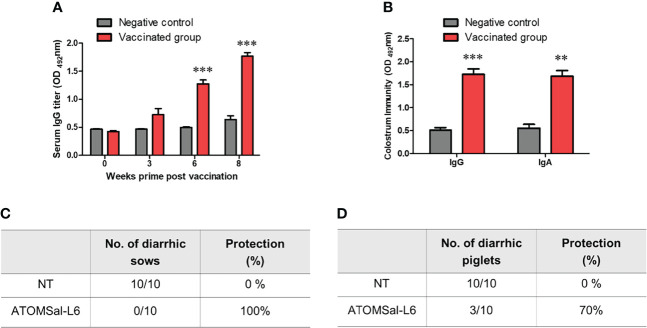
Analysis of humoral immune responses induced by immunization of ATOMSal-L6 in swine. Pregnant sows (n = 3 per group) were immunized orally with 2 × 10^9^ CFU of ATOMSal-L6 twice at three-week intervals. Sera were collected two weeks after the vaccination and colostrum was collected on day of delivery. **(A)** ST-specific serum IgG and **(B)** ST-specific colostrum IgG and IgA were measured using ELISA. **(C)** Sows (n = 10/group; left panel) and **(D)** piglets (n=10/group; right panel) born from vaccinated sows were orally challenged with 5 × 10^8^ CFU of ST WT and monitored for diarrhea symptoms and survival for 14 days. **P < 0.01, ***P < 0.001, compared to unvaccinated group.

To examine the efficacy of ATOMSal-L6 vaccine (2 × 10^9^ CFU/pig) in pig model, pregnant sows were immunized orally with live ATOMSal-L6 twice at three-week intervals. Sera were collected at 3, 6, and 8 weeks, and *Salmonella*-specific IgG antibodies were measured using ELISA. As shown in **Figure 6A**, the vaccinated group showed the increase IgG levels compared to the unvaccinated group. We also collected colostrum on the day of delivery and observed that *Salmonella-*specific IgG and IgA levels were enhanced in the vaccinated group (**Figure 6B**).

To measure the protective response of ATOMSal-L6 vaccine, vaccinated sows were orally infected with ST WT (5 × 10^8^ CFU/pig) and their diarrheic symptoms were monitored for 14 days. All the vaccinated sows (n=10) were free of diarrheic symptoms, whereas all the unvaccinated sows had severe diarrhea (**Figure 6C**). To determine whether *Salmonella*-specific protective antibodies were delivered from the gilt to the piglet, piglets (n=10) born from vaccinated gilts were infected orally with ST WT (5 × 10^8^ CFU/pig) and their survival rates were monitored. As shown in **Figure 6D**, all piglets from the unvaccinated gilts had severe diarrhea and died at 7 d.p.i, but only 30% of piglets born from vaccinated gilts showed lethal and severe diarrhea at 14 d.p.i. We monitored the surviving piglets for more than 21 d.p.i and found no severe diarrheic symptoms. All these data indicated that ATOMSal-L6 could be a safe and effective live attenuated vaccine in pig.

## Discussion

The *Salmonella* vaccine program in poultry has been successfully implemented to control the prevalence of human Salmonellosis in the UK (50, 51), and mass vaccine administration to economically important animals is considered the best strategy to prevent transmission of *Salmonella* to animals and humans. However, due to the emergence of new serotypes and multi-drug resistant *Salmonella* worldwide (12, 52–54), more effective and broad-spectrum *Salmonella* vaccines are being developed. Unlike conventional inactivated vaccines, live attenuated vaccines could induce life-long immunity through one or two doses by activating multifunctional cellular immune responses (22, 55, 56). Nevertheless, this type of vaccine has not been widely used against bacteria because it could cause diseases in immunocompromised individuals and the vaccine could potentially re-acquire its pathogenicity by reverting the mutation (57, 58). In addition, rapidly developing the vaccines against newly emerging serotypes or new pathogens has proven difficult. In this study, we introduced a technology to rapidly develop a live attenuated *Salmonella* vaccine, ATOMSal-L6, using R-MET that can be attenuated by accelerating mutation. In addition, because R-MET technology can introduce various forms of mutations (deletion, insertion, SNP), it will be possible to solve the problem of current live vaccines that re-acquired pathogenicity by genetic revertant (40, 59, 60).Due to these mutations, ATOMSal-L6 differed in biochemical properties from its parent strain. For example, it produces more flagellin but less mobility, and cannot be grown at high temperatures (45°C). And it was confirmed that resistance to specific antibiotic resistance was reduced compared to ST WT. The resistance to the aminoglycoside antibiotics did not change significantly, but the resistance to the macrolides antibiotics was reduced more than 3 times compared to the ST WT. This change in antibiotic resistance will be a good standard for separating and analyzing wild-type and vaccine strain in the clinical samples. All these genetic and biochemical changes might have contributed to the attenuation and immunological properties of ATOMSal-L6.

To the best of our knowledge, compared to UV radiation and chemical mutagens, γ-radiation has not been widely used to induce mutations in vaccine industries because it requires a high-dose radiation facility, and all mutations must be detected and selected painstakingly at the phenotypic level. However, in recent years, new and re-emerging infectious diseases have become prevalent. Using R-MET, which can rapidly and effectively develop live vaccines, might be more attractive (40). In addition, recent advances in large-scale genomic analysis techniques have enabled easy analysis of the effects of radiation and the location of mutations in the bacterial genome. In this study, we screened only 30 colonies after irradiation using R-MET and found several attenuated candidates with significantly reduced screening times compared to UV or chemical mutagens. Overall, it took about 4 weeks to develop ATOMSal-L6, as the colony selection process took about 2–3 days and the *in vitro* and *in vivo* virulence examination took about 2–3 weeks. However, the whole genome sequence with comparative genomics and genetic stabilization tests are time-consuming, often requiring several months to complete. Therefore, a systematic process to speed up these genomic analysis processes must be developed.

Live attenuated Salmonella vaccines must balance attenuation with immunogenicity. In particular, both CD4^+^ and CD8^+^ T cells are highly associated with protection against early infection of *Salmonella (* 61). CD4^+^ T cells might play a central role in acquired immunity against *Salmonella* infection and make an additional important contribution to both CD8^+^ T cell- and B cell-immunities. Therefore, live attenuated *Salmonella* vaccines are preferred over inactivated vaccines that do not have high T-cell immunity. Since ATOMSal-L6 induced protection against ST WT infection by activation of CD4^+^ and CD8^+^ T cells in mice, it is a good vaccine candidate with the balance between high immunogenicity to enable cellular and humoral immune response and sufficiently high attenuation of its virulence. Our previous study showed that inactivated *S.* Gallinarum activated moderate CD4^+^ and CD8^+^ T cell response, but higher Th17 responses (62). It is known that IL-17, increased by Salmonella infection, stimulates intestinal epithelial cells to enhance the production of antimicrobial proteins and chemokines, thereby inhibiting the early invasion of Salmonella bacteria (63, 64). In contrast, ATOMSal-L6 shows no induction of *Salmonella*-specific Th17 response (data not shown) but does high expression of *Salmonella*-specific CD4^+^ and CD8^+^ T cells. We therefore speculate that ATOMSal-L6 may have mutated genes involved in the expression of IL-17-induced antigens during the R-MET process. Therefore, immunization with inactivated *Salmonella* vaccine together or sequentially is another option to increase ATOMSal-L6 efficacy.

ATOMSal-L6 is the first attenuated *Salmonella* vaccine strain developed using R-MET. It is more sensitive to high temperature and showed lower motility compared to its parent strain. In addition, we found 8 SNPs, 3 deletions, 60 insertions, and loss of one bacteriophage upon comparing its genome with its parent strain. Compared to licensed *Salmonella* vaccine strains, its genomic mutations are wide and variable. Although there is no parent strain for comparison with *Salmonella enterica* Serovar Choleraesuis vaccine strain C500 attenuated by chemical mutation, when compared to another WT SC-B67 strain, it was deficient in the *rpoS* gene, a vital transcriptional regulator playing an important role in *Salmonella* infection (65). Salmovac440 developed by IDT Biologika has only 6 SNPs, but lacks the pathogenic plasmid that encodes a number of virulence factors (26, 65, 66). Therefore, the attenuation of Salmovac440 may be due to the amino acid biosynthetic system and other virulence mechanisms involving the lost pathogenic plasmid. In this study, we did not investigate on the degree to which each of these mutations in ATOMSal-L6 affected the virulence attenuation. Thus, to use it as a vaccine strain, accurate biochemical information of ATOMSal-L6 must be acquired, and in particular, additional research on the relationship with the mutation and the virulence must be performed.

In the absence of overt disease, the vaccine strain attenuated in metabolic gene(s) must be metabolically active to reach immune inductive sites and elicit a biologically relevant protective immunity. However, the hyper-attenuation of vaccine strains may result in lower virulence and less effective protective immune responses. Thus, it is necessary to develop a strain that can moderately reduce virulence and induce immunity at a level that does not cause disease. For example, WT05 is the attenuated *S.* Typhimurium vaccine in which the *aroC* gene, involved in aromatic amino acid biosynthesis, and the *ssaV* gene, a component of a Type 3 secretion system (T3SS) apparatus of *Salmonella* pathogenicity island 2 (SPI-2), are deleted. However, this vaccine strain was eliminated through prolonged defecation in healthy volunteers immunized with WT05, thereby failing the phase 1 clinical trial (67, 68). LH1160, a *phoPQ* mutant strain that controls the transcription of multiple genes necessary for intracellular survival, had been tested in phase 1 clinical trials, but an unacceptable fever was reported in two of six volunteers (15, 68, 69). In contrast, CVD1921, which is mutated in the *guaBA* genes that are involved in the biosynthesis of guanine nucleotides and the *clpP* gene affecting flagella expression, was revealed to be notably attenuated with decreased elimination, systemic spread, and clinical disease manifestations in the digestive tract of the non-human primate model (rhesus macaque) used (70, 71). Another advantage of R-MET is that it can be applied to strains that are not sufficiently attenuated to further reduce pathogenicity, allowing it to be used as a vaccine strain. Therefore, further attenuation with R-MET can be attempted in the event of clinically significant safety issues such as those resulting from the use of WT05 and LH1160. In addition, if ATOMSal-L6 has not been sufficiently attenuated, R-MET may be additionally applied. Thus, R-MET will be a very effective and attractive method for live bacterial vaccine development.

## Data Availability Statement

The datasets presented in this study can be found in online repositories. The names of the repository/repositories and accession number(s) can be found below: NCBI SRA Biproject accession no for HJL222 (ST WT): PRJNA844490; accession no for ATOMSal-L6: PRJNA841760.

## Ethics Statement

The animal study was reviewed and approved by Korea Atomic Energy Research Institute.

## Author Contributions

HJJ, AYJ, KBA, SHH, HKJ, JH, and HSS were responsible for conceptualization of the study. HJJ, AYJ, SJB, and JH performed the experiments and analyzed the data. HJJ, KBA, SHH, JYS, and HSS wrote the manuscript. HSS supervised the work. HSS was responsible for funding acquisition. All authors contributed to the article and approved the submitted version.

## Funding

This work was supported in part by the Internal R&D program of KAERI (523210) funded by Ministry of Science and ICT (MIST) and the National Research Foundation of Korea grants 2017M2A2A6A02020925, NRF-2018K2A206023828, and NRF-2020M2A206023828 to HS and NRF-2019M2D3A2060217 to KA.

## Conflict of Interest

Authors SJB and HKJ are employed by CTCVAC.

The remaining authors declare that the research was conducted in the absence of any commercial or financial relationships that could be construed as a potential conflict of interest.

## Publisher’s Note

All claims expressed in this article are solely those of the authors and do not necessarily represent those of their affiliated organizations, or those of the publisher, the editors and the reviewers. Any product that may be evaluated in this article, or claim that may be made by its manufacturer, is not guaranteed or endorsed by the publisher.
