# Supplementary figures and images for: Development of Live Attenuated Salmonella Typhimurium Vaccine Strain Using Radiation Mutation Enhancement Technology (R-MET)
Source: Front Immunol. 2022 Jul 11;13:931052. doi: 10.3389/fimmu.2022.931052 (PMC9310569; doi:10.3389/fimmu.2022.931052)

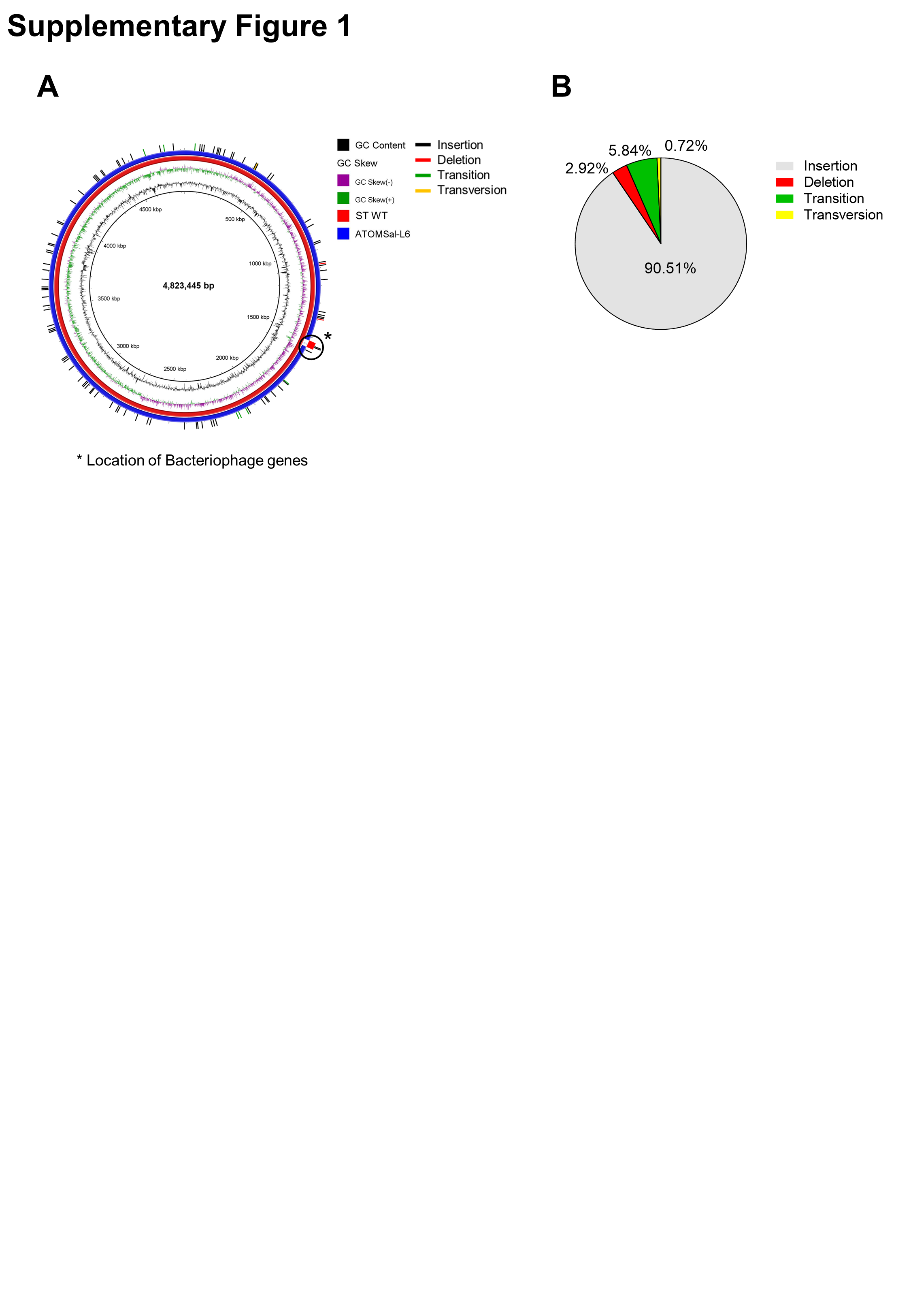

Supplement: Supplementary file 1 [file Image_1.tif]

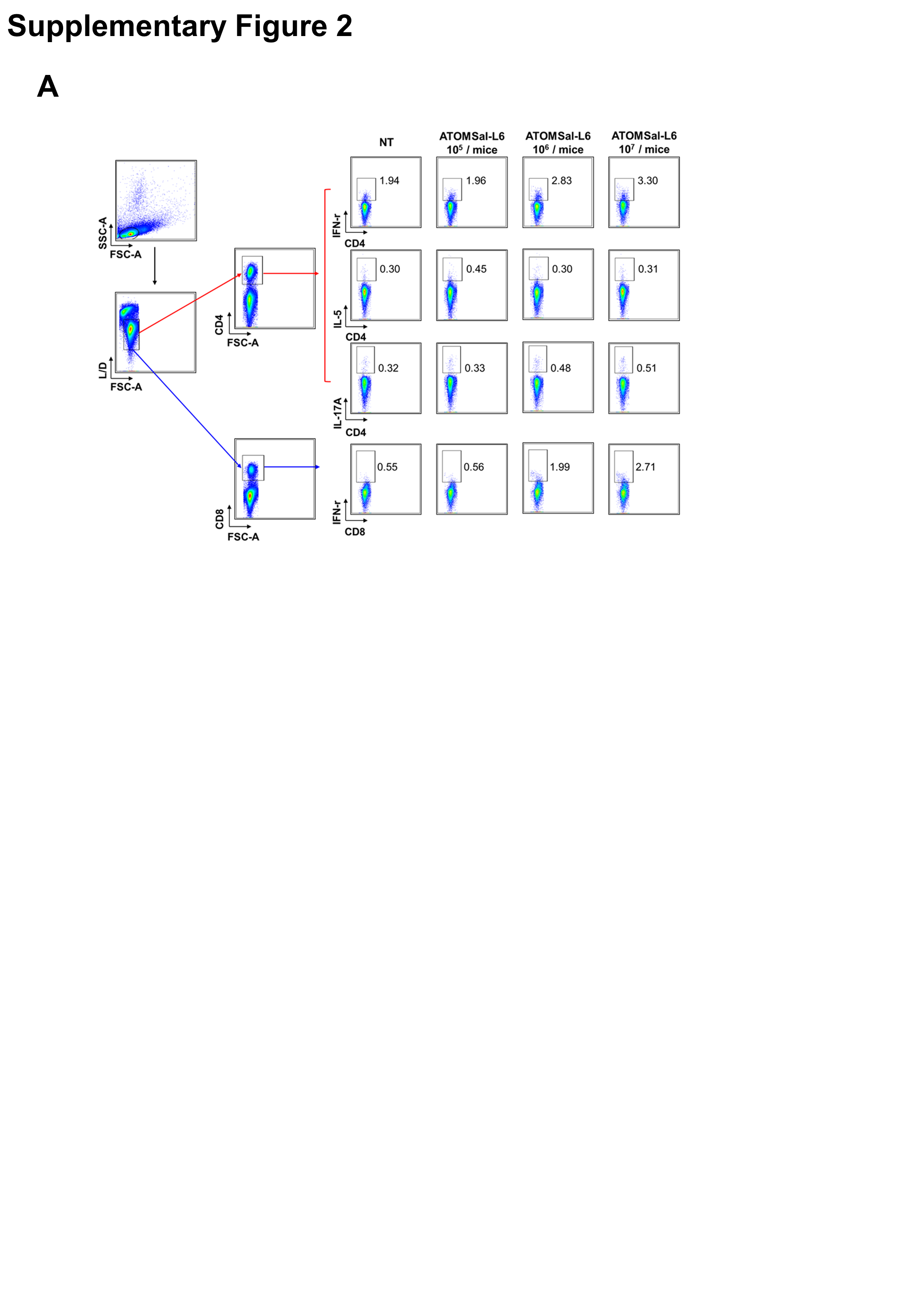

Supplement: Supplementary file 2 [file Image_2.tif]

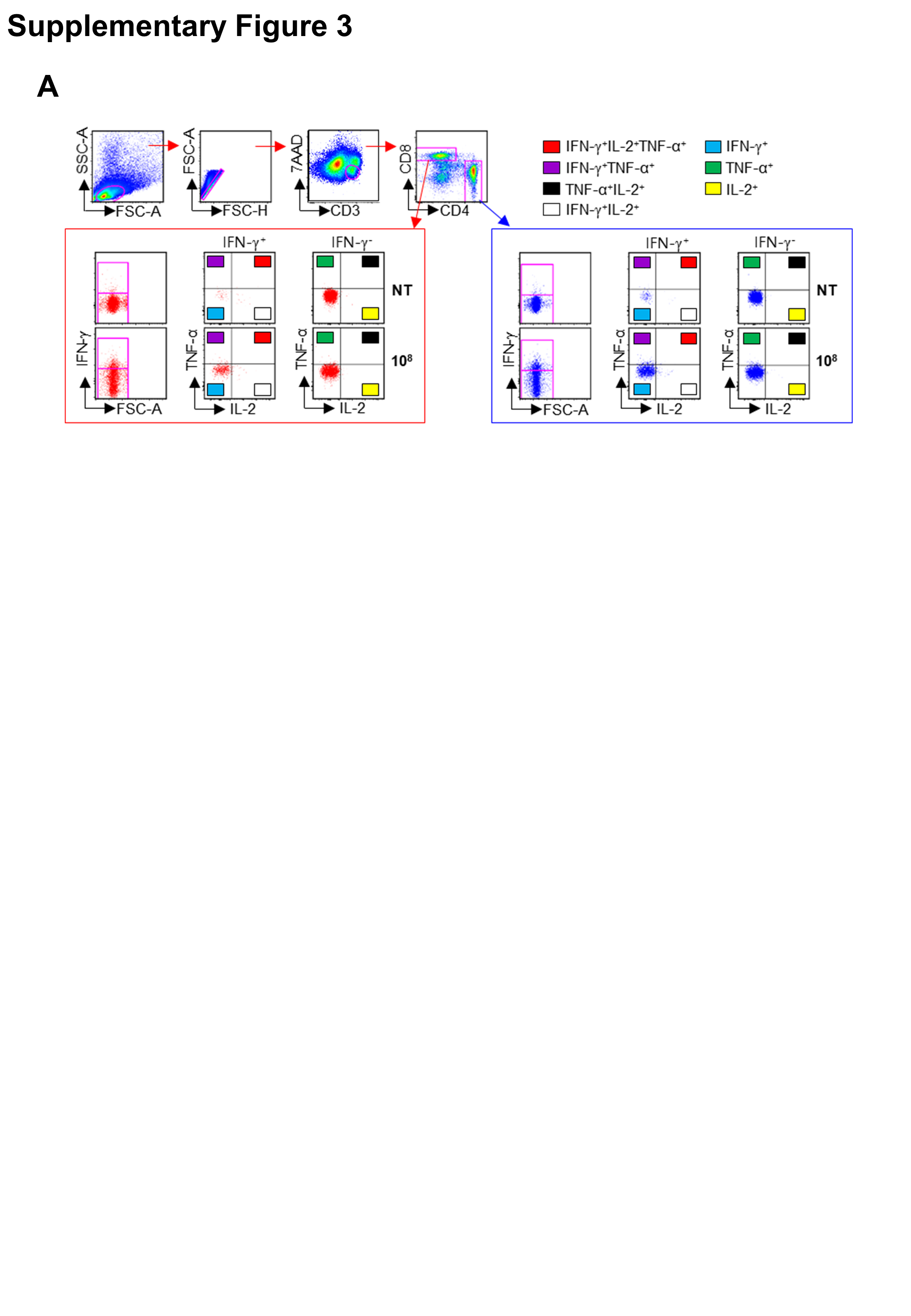

Supplement: Supplementary file 3 [file Image_3.tif]
