# Supplementary material for: Development of Live Attenuated Salmonella Typhimurium Vaccine Strain Using Radiation Mutation Enhancement Technology (R-MET)
Source: Front Immunol. 2022 Jul 11;13:931052. doi: 10.3389/fimmu.2022.931052 (PMC9310569; doi:10.3389/fimmu.2022.931052)
Supplement: Supplementary file 4 [file Table_1.docx]

**Supplementary Table S1.** Biochemical characterization by API 20NE of ST WT and ATOMSal-L6

| **Substrate** | | **ST WT** | **ATOMSal-L6** |
| --- | --- | --- | --- |
| Reduction of | |  |  |
| 1 | Nitrates to mitrites | **+** | **+** |
| 2 | Nitrates to nitrogen | **-** | **-** |
| Indole production | | **-** | **-** |
| Fermentation of glucose | | **+** | **+** |
| Hydrolysis of | |  |  |
| 1 | Esculin | **w** | **w** |
| 2 | Gelatin | **-** | **-** |
| Assimilation of | |  |  |
| 1 | Glucose | **+** | **+** |
| 2 | Arabinose | **+** | **+** |
| 3 | Mannose | **+** | **+** |
| 4 | Mannitol | **+** | **+** |
| 5 | N-acetyl-glucosamine | **+** | **+** |
| 6 | Maltose | **+** | **+** |
| 7 | Gluconate | **+** | **+** |
| 8 | Caprate | **-** | **-** |
| 9 | Adipate | **-** | **-** |
| 10 | Malate | **+** | **+** |
| 11 | Citrate | **+** | **+** |
| 12 | Penyl-acetate | **-** | **-** |

Abbreviations: +, positive; -, negative; W, weakly positive

**Supplementary Table S2.** Biochemical characterization by ZYM of ST WT and ATOMSal-L6

| **Substrate** | | **ST WT** | **ATOMSal-L6** |
| --- | --- | --- | --- |
| Enzyme activity | |  |  |
| 1 | Urease | **-** | **-** |
| 2 | Arginine dihydrolase | **+** | **+** |
| 3 | Alkaline phosphatase | **+** | **+** |
| 4 | Esterase (C4) | **-** | **+** |
| 5 | Esterase lipase (C8) | **+** | **+** |
| 6 | Lipase (C14) | **+** | **+** |
| 7 | Leucine arylamidase | **+** | **+** |
| 8 | Valine arylamidase | **-** | **-** |
| 9 | Cystine arylamidase | **-** | **-** |
| 10 | Trypsin | **-** | **-** |
| 11 | α-Chymotrypsin | **-** | **-** |
| 12 | Acid phosphatase | **+** | **+** |
| 13 | Naphthol-AS-BI-phosphohydrolase | **+** | **+** |
| 14 | α-Galactosidase | **-** | **-** |
| 15 | β-Galactosidase | **-** | **-** |
| 16 | β-Glucuronidase | **-** | **-** |
| 17 | α-Glucosidase | **-** | **-** |
| 18 | β-Glucosidase | **-** | **-** |
| 19 | N-Acetyl-β-glucoaminidase | **-** | **-** |
| 20 | α-Mannosidase | **-** | **-** |
| 21 | α-Fucosidase | **-** | **-** |

Abbreviations: +, positive; -, negative; W, weakly positive

**Supplementary Table S3.** Biochemical characterization by CH50 of ST WT and ATOMSal-L6

| **Substrate** | | **ST WT** | **ATOMSal-L6** | **Substrate** | | | **ST WT** | **ATOMSal-L6** |
| --- | --- | --- | --- | --- | --- | --- | --- | --- |
| Oxidation of | |  |  | Oxidation of | |  | |  |
| 1 | Glycerol | **-** | **-** | 26 | Salicin | **-** | | **-** |
| 2 | Erythritol | **-** | **-** | 27 | Cellobiose | **-** | | **-** |
| 3 | D-Arabinose | **-** | **-** | 28 | Maltose | **-** | | **-** |
| 4 | L-Arabinose | **w** | **+** | 29 | Lactose | **-** | | **-** |
| 5 | D-Ribose | **-** | **w** | 30 | Melibiose | **-** | | **w** |
| 6 | D-Xylose | **-** | **-** | 31 | Sucrose | **-** | | **-** |
| 7 | L-Xylose | **-** | **-** | 32 | Trehalose | **w** | | **w** |
| 8 | D-Adonitol | **-** | **-** | 33 | Inulin | **-** | | **-** |
| 9 | Methyl β-xyloside | **-** | **-** | 34 | Melezitose | **-** | | **-** |
| 10 | D-Galactose | **+** | **+** | 35 | Raffinose | **-** | | **-** |
| 11 | D-Glucose | **+** | **+** | 36 | Starch | **-** | | **-** |
| 12 | D-Fructose | **+** | **+** | 37 | Glycogen | **-** | | **-** |
| 13 | D-Mannose | **-** | **+** | 38 | Xylitol | **-** | | **-** |
| 14 | L-Sorbose | **-** | **-** | 39 | Gentiobiose | **-** | | **-** |
| 15 | L-Rhamnose | **-** | **w** | 40 | Turanose | **-** | | **-** |
| 16 | Dulcitol | **-** | **-** | 41 | D-Lyxose | **-** | | **-** |
| 17 | Inositol | **-** | **-** | 42 | D-Tagatose | **-** | | **-** |
| 18 | D-Mannitol | **-** | **-** | 43 | D-Fucose | **-** | | **-** |
| 19 | D-Sorbitol | **-** | **-** | 44 | L-Fucose | **-** | | **-** |
| 20 | Methyl α-mannoside | **-** | **-** | 45 | D-Arabitol | **-** | | **-** |
| 21 | Methyl α-glucoside | **-** | **-** | 46 | L-Arabitol | **-** | | **-** |
| 22 | N-Acetylglucosaamine | **-** | **-** | 47 | Gluconate | **-** | | **-** |
| 23 | Amygdalin | **-** | **-** | 48 | 2-ketogluconate | **-** | | **-** |
| 24 | Arbutin | **-** | **-** | 49 | 5-ketogluconate | **-** | | **-** |
| 25 | Esculin | **-** | **-** |  |  |  | |  |

Abbreviations: +, positive; -, negative; W, weakly positive

**Supplementary Table S4.** MIC test results of ST WT and ATOMSal-L6

|  | KAN (μg/ml) | TET (μg/ml) | ERM (μg/ml) | AMP (μg/ml) | CFR (μg/ml) | TMP (μg/ml) | GEN (μg/ml) | AMC (μg/ml) | AMK (μg/ml) | STR (μg/ml) | SPT (μg/ml) | LIN (μg/ml) | CLI (μg/ml) | TOB (μg/ml) |
| --- | --- | --- | --- | --- | --- | --- | --- | --- | --- | --- | --- | --- | --- | --- |
| ST WT | 22.9 | 206 | 68.6 | 15.2 | 0.5 | <0.5 | 6.2 | 1.4 | 12.3 | 111 | 37.0 | 333 | 333 | 4.1 |
| ATOMSal-L6 | 22.9 | 68.9 | 22.9 | 1.7 | 0.5 | <0.5 | 2.1 | 0.5 | 4.1 | 37.0 | 4.1 | 37.0 | 37.0 | 4.1 |

Abbreviations: KAN, kamamycin; TET, tetracycline; ERM, erythromycin; AMP, ampicillin; CFR, Cefadroxil; TMP, trimethoprim; GEN, gentamicin; AMC, amoxicilllin; AMK, amikacin; STR, streptomycin; SPT, spectinomycin; LIN, lincomycin; CLI, clindamycin; TOB, tobramycin.

**Supplementary Table S5.** Location of mutations in ATOMSal-L6 genome

|  | **Gene ID** | **Gene Name** | **Position** | **ST WT** | **ATOMSal-L6** | **Mutation** | **Type**  **(Effect on the amino acid)** |
| --- | --- | --- | --- | --- | --- | --- | --- |
| 1 | ST WT_1_00068 | trpB | 69952 | G | A | Transition | Silent |
| 2 |  | NCR | 93846 | TCCCCC | TCCCCCC | Insertion | - |
| 3 |  | NCR | 99388 | TCCCC | TCCCCC | Insertion | - |
| 4 | ST WT_1_00106 | narK | 109736 | TCCCCCC | TCCCCCCC | Insertion | Frame shift |
| 5 | ST WT_1_00162 | yoaA | 166311 | TCCCC | TCCCCC | Insertion | Frame shift |
| 6 | ST WT_1_00182 | ribZ | 186453 | TCCCC | TCCCCC | Insertion | Frame shift |
| 7 |  | NCR | 197166 | TCCCCC | TCCCCCC | Insertion | - |
| 8 |  | NCR | 209696 | CGGGG | CGGGGG | Insertion | - |
| 9 | ST WT_1_00231 | mepM | 232152 | TCCCC | TCCCCC | Insertion | Frame shift |
| 10 | ST WT_1_00263 | motB | 266648 | TCCCCCC | TCCCCCCC | Insertion | Frame shift |
| 11 |  | NCR | 271422 | CGGGGGG | CGGGGGGG | Insertion | - |
| 12 | ST WT_1_00351 | cobS | 345747 | GCCCCC | GCCCCCC | Insertion | Frame shift |
| 13 | ST WT_1_00418 | rfbM | 410699 | AGGGGG | AGGGGGG | Insertion | Frame shift |
| 14 | ST WT_1_00418 | rfbM | 411002 | T | A | Transversion | Mis-sense |
| 15 | ST WT_1_00427 | ascD | 421048 | TCCCCCC | TCCCCCCC | Insertion | Frame shift |
| 16 | ST WT_1_00498 | yehX | 504717 | CGGGGG | CGGGGGG | Insertion | Frame shift |
| 17 | ST WT_1_00525 | galS | 534956 | GCCCC | GCCCCC | Insertion | Frame shift |
| 18 | ST WT_1_00819 | acrB | 844222 | CGGGG | CGGGGG | Insertion | Frame shift |
| 19 |  | NCR | 868896 | ACCCCC | ACCCCCC | Insertion | - |
| 20 | ST WT_1_00905 | murQ | 964384 | CGGGGG | CGGGGGG | Insertion | Frame shift |
| 21 |  | NCR | 978034 |  |  | Insertion | - |
| 22 | ST WT_1_01024 | bapA | 1075238 | GCCCCC | GCCCCCC | Insertion | Frame shift |
| 23 | ST WT_1_01026 | apxIB | 1088696 | CAAAAAAAAA | CAAAAAAAA | Deletion | Frame shift |
| 24 | ST WT_1_01031 | yidE | 1094490 | AGGGGG | AGGGGGG | Insertion | Frame shift |
| 25 |  | NCR | 1125501 | AGGGGG | AGGGGGG | Insertion | - |
| 26 |  | NCR | 1128306 | AC | ACC | Insertion | - |
| 27 |  | NCR | 1128508 | ATTTTT | ATTTTTT | Insertion | - |
| 28 |  | NCR | 1128565 | GC | GCC | Insertion | - |
| 29 |  | NCR | 1128751 | GTT | GTTT | Insertion | - |
| 30 |  | NCR | 1128775 | TCC | TCCC | Insertion | - |
| 31 |  | NCR | 1128962 | CA | CCAA | Insertion? | - |
| 32 |  | NCR | 1129023 | ATT | ATTT | Insertion | - |
| 33 |  | NCR | 1129051 | CGGG | CGGGG | Insertion | - |
| 34 |  | NCR | 1129157 | ACC | ACCC | Insertion | - |
| 35 |  | NCR | 1129238 | CTT | CTTT | Insertion | - |
| 36 | ST WT_1_01107 | emrB | 1176927 | TGGGGG | TGGGGGG | Insertion | Frame shift |
| 37 | ST WT_1_01283 | Hyphothetical gene | 1354871 | GCCCC | GCCCCC | Insertion | Frame shift |
| 38 | ST WT_1_01292 | lplT | 1370267 | GCCCC | GCCCCC | Insertion | Frame shift |
| 39 | ST WT_1_01309 | ygeA | 1389297 | TCCCCCC | TCCCCCCC | Insertion | Frame shift |
| 40 |  | NCR | 1389428 | AGGGGG | AGGGGGG | Insertion | - |
| 41 | ST WT_1_01318 | rcnA | 1398175 |  |  | Deletion |  |
| 42 | ST WT_1_01467 | dctM | 1547912 | GCCCC | GCCCCC | Insertion | Frame shift |
| 43 |  | NCR | 1566505 | TCCCC | TCCCCC | Insertion | - |
| 44 |  | NCR | 1589946 | GCCCC | GCCCCC | Insertion | - |
| 45 | ST WT_1_01707 | Fmt | 1791961 | T | C | Transition | Mis-sense |
| 46 | ST WT_1_01713 | Hyphothetical gene | 1796252 | AGGGG | AGGGGG | Insertion | Frame shift |
| 47 | ST WT_1_01778 | bigA | 1843111 | ACCCCCC | ACCCCCC | Insertion | Frame shift |
| 48 | ST WT_1_01815 | malt | 1891701 | TGGGG | TGGGGG | Insertion | Frame shift |
| 49 |  | NCR | 1942076 | CGGGG | CGGGGG | Insertion | - |
| 50 |  | NCR | 1946897 | GCCCC | GCCCCC | Insertion | - |
| 51 |  | NCR | 1974867 | TCCCCC | TCCCCCC | Insertion | - |
| 52 |  | NCR | 2049229 | GCCCCC | GCCCCCC | Insertion | - |
| 53 | ST WT_1_01962 | malS | 2064783 | C | T | Transition | Mis-sense |
| 54 | ST WT_1_01998 | gpsA | 2109247 | C | T | Transition | Mis-sense |
| 55 | ST WT_1_02016 | rfaI | 2127888 | T | C | Transition | Mis-sense |
| 56 | ST WT_1_02122 | torA | 2236636 | GCCCCC | GCCCCCC | Insertion | Frame shift |
| 57 | ST WT_1_02134 | dgoD | 2250500 | TGGGGG | TGGGGGG | Insertion | Frame shift |
| 58 |  | NCR | 2271158 | TCCCCC | TCCCCCC | Insertion | - |
| 59 |  | NCR | 2322348 | ACCCC | ACCCCC | Insertion | - |
| 60 | ST WT_1_02245 | dapF | 2354695 | TCCCCCCC | TCCCCCCCC | Insertion | Frame shift |
| 61 |  | NCR | 2355037 | CGGGGG | CGGGGG | Insertion | - |
| 62 | ST WT_1_02298 | engB | 2423069 | TCCCC | TCCCCC | Insertion | Frame shift |
| 63 | ST WT_1_02471 | Hyphothetical gene | 2601513 | CGGGGG | CGGGGGG | Insertion | Frame shift |
| 64 | ST WT_1_02498 | Hyphothetical gene | 2634855 | GCCCCCC | GCCCCCCC | Insertion | Frame shift |
| 65 | ST WT_1_02545 | alr | 2682867 | TGGGG | TGGGGG | Insertion | Frame shift |
| 66 |  | NCR | 2751834 | AGGGGGG | AGGGGGGG | Insertion | - |
| 67 |  | NCR | 2791055 | ACCCC | ACCCCC | Insertion | - |
| 68 | ST WT_1_02643 | mscM | 2806645 | CGGGG | CGGGGG | Insertion | Frame shift |
| 69 |  | NCR | 2845108 | GCCCCC | GCCCCCC | Insertion | - |
| 70 |  | NCR | 2945460 | GCCCCC | GCCCCCC | Insertion | - |
| 71 |  | NCR | 2974812 | TCCCC | TCCCCC | Insertion | - |
| 72 |  | NCR | 2978501 | TGGGGGG | TGGGGGG | Insertion | Frame shift (ST WT_1_02823 start codon lost) |
| 73 | ST WT_1_02833 | agaC | 3010207 | GCCCC | GCCCCC | Insertion | Frame shift |
| 74 | ST WT_1_02848 | Hyphothetical gene | 3024787 | AGGGGGGG | AGGGGGGGG | Insertion | Frame shift |
| 75 |  | NCR | 3024919 | ACCCC | ACCCCC | Insertion | - |
| 76 |  | NCR | 3025017 | AGGGGGGG | AGGGGGGGG | Insertion | - |
| 77 | ST WT_1_02859 | rssA | 3031475 | CGGGGG | CGGGGGG | Insertion | Frame shift |
| 78 | ST WT_1_02889 | sthD | 3063700 | AGGGG | AGGGGG | Insertion | Frame shift |
| 79 | ST WT_1_02960 | Hyphothetical gene | 3146839 | CGGGGGG | CGGGGGGG | Insertion | Frame shift |
| 80 |  | NCR | 3193200 | CGGGGG | CGGGGGG | Insertion | - |
| 81 | ST WT_1_03009 | ilvl | 3209087 | CGGGG | CGGGGG | Insertion | Frame shift |
| 82 |  | NCR | 3211528 | CGGGGG | CGGGGGG | Insertion | - |
| 83 |  |  | 3363043 | TCCCC | TCCCCC | Insertion | Frame shift |
| 84 | ST WT_1_03150 | dmlR | 3367754 | GCC | GCCC | Insertion | Frame shift |
| 85 | ST WT_1_03160 | impA | 3377190 | TGGGGG | TGGGGGG | Insertion | Frame shift |
| 86 | ST WT_1_03165 | tagK | 3381621 | TCCCCCC | TCCCCCCC | Insertion | Frame shift |
| 87 | ST WT_1_03187 | tssI | 3409015 | CGGGGGG | CGGGGGGG | Insertion | Frame shift |
| 88 | ST WT_1_03295 | stdD | 3493340 | ACCC | ACCCC | Insertion | Frame shift |
| 89 | ST WT_1_03310 | bepF | 3511129 | CGGGGG | CGGGGGG | Insertion | Frame shift |
| 90 | ST WT_1_03355 | sbcD | 3562896 | CGGGGGG | CGGGGGGG | Insertion | Frame shift |
| 91 | ST WT_1_03396 | Hyphothetical gene | 3603952 | CGGGGG | CGGGGGG | Insertion | Frame shift |
| 92 | ST WT_1_03403 | ampG | 3611779 | CGGG | CGGGG | Insertion | Frame shift |
| 93 | ST WT_1_03408 | clpX | 3618343 | AGGGGG | AGGGGGG | Insertion | Frame shift |
| 94 | ST WT_1_03416 | cof | 3627993 | TCCCC | TCCCCC | Insertion | Frame shift |
| 95 | ST WT_1_03447 | Aes | 3661709 | AGGGGG | AGGGGGG | Insertion | Frame shift |
| 96 | ST WT_1_03501 | fimA | 3717677 | TCCCC | TCCCCC | Insertion | Frame shift |
| 97 | ST WT_1_03526 | lip1 | 3740459 | AGGGGG | AGGGGGG | Insertion | Frame shift |
| 98 | ST WT_1_03594 | rlpA | 3813256 | CGGGG | CGGGGG | Insertion | Frame shift |
| 99 | ST WT_1_03616 | Hyphothetical gene | 3837690 | AGGGG | AGGGGG | Insertion | Frame shift |
| 100 |  | NCR | 3858196 | AGGGG | AGGGGG | Insertion | - |
| 101 |  | NCR | 3862274 | TCCCC | TCCCCC | Insertion | - |
| 102 | ST WT_1_03691 | odhA | 3915381 | AGGGGG | AGGGGGG | Insertion | Frame shift |
| 103 | ST WT_1_03752 | bioF | 3974695 | CGGGGG | CGGGGGG | Insertion | Frame shift |
| 104 | ST WT_1_03763 | ybhL | 3986565 | CGGGGG | CGGGGGG | Insertion | Frame shift |
| 105 | ST WT_1_03893 | focA | 4128176 | ACCCC | ACCCCC | Insertion | Frame shift |
| 106 |  | NCR | 4182555 | AGGGGGG | AGGGGGGG | Insertion | - |
| 107 |  | NCR | 4294382 | TCCCCC | TCCCCCC | Insertion | - |
| 108 |  | NCR | 4294643 | AGGGGGG | AGGGGGGG | Insertion | - |
| 109 |  | NCR | 4294748 | TCCCCC | TCCCCCC | Insertion | - |
| 110 | ST WT_1_04068 | ymdA | 4303075 | AGGGGGGGGG | AGGGGGGGGGG | Insertion | Frame shift |
| 111 | ST WT_1_04078 | Hyphothetical gene | 4314089 | ACCC | ACCCC | Insertion | Frame shift |
| 112 | ST WT_1_04119 | fabB | 4351023 | AGGGGG | AGGGGGG | Insertion | Frame shift |
| 113 |  | NCR | 4356381 | TCCCCC | TCCCCCC | Insertion | - |
| 114 | ST WT_1_04125 | ptsG | 4356797 | CGGGGG | CGGGGGG | Insertion | Frame shift |
| 115 | ST WT_1_04137 | ycfS | 4368710 | CGGGGG | CGGGGGG | Insertion | Frame shift |
| 116 | ST WT_1_04215 | ydjA | 4445216 | CGGGGGG | CGGGGGGG | Insertion | Frame shift |
| 117 | ST WT_1_04226 | astE | 4458181 | AGGGG | AGGGGG | Insertion | Frame shift |
| 118 | ST WT_1_04260 | btuC | 4489777 | CGGGGG | CGGGGGG | Insertion | Frame shift |
| 119 | ST WT_1_04292 | iscS | 4527001 | AGGGG | AGGGGG | Insertion | Frame shift |
| 120 |  | NCR | 4606455 | T | C | Transition | - |
| 121 | ST WT_1_04465 | Fmo | 4693085 | AGGGG | AGGGGG | Insertion | Frame shift |
| 122 | ST WT_1_04472 | Hyphothetical gene | 4698466 | CGGGGG | CGGGGGG | Insertion | Frame shift |
| 123 | ST WT_1_04487 | maeA | 4716677 | T | C | Transition | Silent |
| 124 | ST WT_1_04528 | ydcO | 4766978 | TCCCCCC | TCCCCCCC | Insertion | Frame shift |
| 125 | ST WT_2_00009 | iucB | 10752 | CGGGGGGG | CGGGGGGGG | Insertion | Frame shift |
| 126 | ST WT_2_00050 | traI | 47211 | AGGGG | AGGGGG | Insertion | Frame shift |
| 127 | ST WT_2_00051 | traI | 48032 | TCCCCCC | TCCCCCCC | Insertion | Frame shift |
| 128 | ST WT_2_00053 | traD | 48544 | TCCCCC | TCCCCCC | Insertion | Frame shift |
| 129 |  | NCR | 84082 | CGGGG | CGGGGG | Insertion | - |
| 130 | ST WT_2_00096 | Hyphothetical gene | 84575 | TAGCAG |  | deletion | Frame shift |
| 131 |  | NCR | 85394 | TCCCC | TCCCCC | Insertion | - |
| 132 |  | NCR | 85410 | CGGGGG | CGGGGGG | Insertion | - |
| 133 | ST WT_2_00099 | Hyphothetical gene | 86919 | GCCCC | GCCCCC | Insertion | Frame shift |
| 134 |  | NCR | 94969 | G | A | Transition | - |
| 135 |  | NCR | 95016 | TCC | TCCC | Insertion | - |
| 136 |  | NCR | 95031 | AGAC | AGACCGAC | Insertion | - |
| 137 |  | NCR | 103626 | GCCCC | GCCCCC | Insertion |  |

Abbreviations: NCR, Non Coding Region
